# Low-Carbon Path Transformation for Different Types of Enterprises under the Dual-Carbon Target

**DOI:** 10.3390/ijerph20065167

**Published:** 2023-03-15

**Authors:** Qibao Shi, Weina Xu

**Affiliations:** School of Economics and Management, Shanghai University of Political Science and Law, Shanghai 201701, China

**Keywords:** dual-carbon target, low-carbon path, path transformation, multi-objective optimization, idea point method

## Abstract

Under pressure from the environment and resources, emission peak and carbon neutrality have rapidly become a global issue. The optimization of the ecological goal should be in line with the energy target. In most instances, however, the economic and the ecological goals cannot be unified. This paper establishes a multi-objective optimization model that maximizes the economic benefit of enterprises and the ecosystem activity of the government in the meantime. The idea point method is used in solving this multi-objective optimization problem in the form of a single-objective optimization problem. The numerical experiment documents four types of Chinese enterprises, which are primary resources, industrial manufacturing, public services and commercial consumption. Some management insights are summarized at the end, such as the cores of achieving high-quality and low-carbon development are industrial manufacturing and public services in China.

## 1. Introduction

Climate change is a common challenge faced by human society today, which has a significant impact on the global natural ecosystem, and also brings a severe test to human survival and development. China, as a big country of greenhouse gas emissions, has been actively doing a good job in reducing emissions and taking the road of green- and low-carbon development. China’s carbon dioxide emissions rose from 9.24 billion tons in 2014 to 9.83 billion tons in 2019, an average annual growth rate of 1.2 percent [1,2]. Therefore, the realization of the “double carbon” goal of the country is a broad and profound change, and China will make great efforts for it. As the basic economic organization of society, enterprises are bound to be the backbone of realizing the goal of double carbon. To help the realization of peak carbon dioxide emissions and carbon neutrality, enterprises should optimize the layout from the aspects of industrial layout, resource endowment characteristics and the introduction of advanced technology, focus on the realization of intelligent service operations, and realize the development path of energy saving and low carbon through the Internet of Things, digitization and other measures.

With the rapid development of modern industrialization, people’s living standards have improved to a large extent. However, the consumption of large amounts of fossil energy has also led to a sharp increase in carbon dioxide emissions, with around 73% of global carbon emissions originating from the energy sector. In its report “Global Energy Review: CO_2_ Emissions 2021”, the International Energy Agency states that global CO_2_ emissions from the energy sector reach 36.3 billion tons in 2021, up 6% year-on-year [3,4]. Clearly, with the increasing concentration of greenhouse gases, the issue of climate change has become one of the serious challenges faced by all mankind in the 21st century. Controlling carbon emissions and developing a green economy have become the consensus of all countries in the world, and countries around the world have set corresponding carbon emission reduction targets and taken climate change response actions, such as carbon cap and other emission reduction policies. Under pressure from the environment and resources, carbon peaking and carbon neutrality are gradually becoming global issues. Compared to Europe and the United States, China is still in an economic upturn, and the interval between achieving carbon peaking and carbon neutrality is relatively short, so it is difficult to achieve the vision of carbon neutrality [5]. At present, China’s economy has shifted from a stage of rapid growth to a stage of high-quality development, and low-carbon, environmentally friendly and green development is a necessary part of high-quality development.

Under double pressure from the environment and resources, carbon peak and carbon neutrality have gradually become a global issue [6,7,8]. At present, China’s economy has converted to a high-quality development from a high-speed growth. Low carbon and environment protection are necessary steps of high-quality development. As policies and actions on environmental protection and tackling climate change continue to deepen, China’s attention on green development will reach an unprecedented new altitude. The key to promoting green development lies in green technological innovation, which is an important guarantee for achieving the goal of carbon neutrality. In 2019, the “Guiding Opinions on Constructing a Market-Oriented Green Technology Innovation System”, jointly released by the National Development and Reform Commission and the Ministry of Science and Technology, clearly defined green technology as emerging technologies that reduce consumption, reduce pollution, improve ecology, promote the construction of ecological civilization and achieve harmonious coexistence between humans and nature. This includes energy conservation and environmental protection, clean production, clean energy, ecological protection and restoration, urban and rural green infrastructure, and ecological agriculture and other fields, covering technologies in product design, production, consumption, recycling and other aspects [9].

In the short term, green innovation is urgently needed to properly handle the contradiction between economic transformation and development, post-epidemic economic recovery and carbon emission policy constraints. In the long term, maintaining low-carbon and decarbonizing development depends on green technology, and the key to enhance China’s competitiveness in the international carbon market lies in green technology innovation. In addition, with the continuous improvement of consumers’ environmental awareness, various green technology products are favored by consumers, which encourages enterprises to adjust their business strategies through green technology innovation activities. Therefore, it is particularly necessary for both the government and enterprises to establish a new development concept of innovation, coordination, eco friendliness, openness and sharing, and to seek an effective means to achieve the coordinated development of economy and the environment.

In addition, the government work report of the 2022 Chinese National People’s Congress emphasized the orderly promotion of carbon peaking and carbon neutrality, and the promotion of research, development and application of green- and low-carbon technologies. The China Green Technology Innovation Index Report (2021) pointed out that green technology, as one of the main green innovation vehicles and manifestations, has both commercial characteristics of improving production efficiency and enterprise competitiveness, and social characteristics of environmental protection and energy conservation and emission reduction, and is a fundamental way to solve the dilemma of economic development, energy conservation and emission reduction.

The optimization of the ecological goal should be in line with the energy target. In most instances, the economic target and the ecological goal cannot be unified. There is a compromise proposal between these two goals. This paper establishes a multi-objective optimization model that minimizes the energy system sensitivity and maximizes ecosystem activity in the meantime. We summarize the major contributions of this paper as follows:

- Firstly, from the original single economic goal to the establishment of a multi-objective optimization model for depicting the contradiction between economy and ecology, this paper would like to find an equilibrium state in the low-carbon transformation.
- The development path of enterprises’ low-carbon transformation is given, and the portfolio strategy model of enterprise low-carbon transformation is conducted.
- Chinese enterprises are divided into four categories: primary resources, industrial manufacturing, public services and commercial consumption. The simulation experiment of the low-carbon development path for four types of enterprises are given. Some management insights are summarized at the end, such as the cores of achieving high-quality and low-carbon development within industrial manufacturing and public services in China.

## 2. Literature Review

Our research is closely related to three streams of literature, including the multi-objective optimization model of enterprises’ low-carbon development, strategy choices between economic benefits of enterprises and ecological efficiency of the government low-carbon development, as well as portfolio strategies for low-carbon corporate development.

Concerning the research of the single-objective optimization model and multi-objective optimization model for the low-carbon development of enterprises, McKinnon and Piecyk review the measurement methods of carbon emissions from road transportation in the UK. They also put forward some suggestions on the measurement methods and ranges of carbon emissions according to the actual situation in the United Kingdom, which has referential significance on the calculation of carbon emissions in road transportation in other countries [10]. Based on the premise of a linear relationship between carbon emissions and orders, Haassi et al. study the ordering strategy under the carbon emission constraint policy [11]. Rezaee et al. establish a two-stage stochastic programming model for the green supply chain of carbon environment trading to minimize the carbon emissions on the whole supply chain [12]. Considering the carbon transfer cost and carbon holding cost in the supply chain, Tao et al. establish a multi-stage dynamic programming model of the green supply chain subjecting to carbon footprint constraints to study the influence of carbon transfer and hold cost on inventory control strategy and supply chain coordination [13]. Orlov et al. construct a multi-sector static CGE model to study the economic impact of the implementation of carbon tax in Russia, and to simulate the impact of replacing carbon tax with labor tax on carbon emissions and residents’ welfare [14,15]. The study of Chertow and Lombardi [16], taking the Guayama area of Puerto Rican as an example, evaluates the industrial symbiosis from the aspects of the environmental benefit of enterprise production, the operating income and profit level of enterprise operation, and the supervision of enterprise by the government as an external force, etc. The environmental impact level is measured by measuring the reduced pollution emissions of the local enterprise community. 

There are several low-carbon path transformations. Chen finds that digital transformation is an important breakthrough technology to deal with the negative effects of low-carbon development [17,18]. Chen et al. show that an increase in one standard deviation in the provincial debt-to-GDP ratio results in a 3.08% decline in the level of corporate green innovation [19]. Wang et al. and Dong et al. use the carbon trading mechanism to help thermal power enterprises to obtain long-term profits through frontier analysis and Porter hypothesis analysis, respectively [20,21]. By taking into account the dynamic changes in electricity demand to cope with the changes brought by low-carbon policies and socio-economic development, Matthew et al. construct an integrated system dynamic model covering 2005–2050, taking St. Michael Island of the Azores Islands as an example [22]. This paper analyzes the electricity demand by introducing tourism development, energy efficiency, and electric vehicle expansion, and points out that the expansion of electric vehicles can effectively promote the green development of electric power. By studying the present situation of low-carbon development in Argentina, Colombia, and Brazil, Sheinbaum et al. found that by increasing the proportion of clean energy, such as hydropower and wind power, the energy consumption intensity of the manufacturing industry can be reduced, which is beneficial to its low-carbon development [23]. Panigrahi et al. summarize the application of common evolutionary algorithms in economic load and environment problems and introduce the advantages of heuristic algorithms in economic load distribution optimization [24]. The optimization method in economic load dispatching is summarized by Huneault and Galiana, and its significance for power system optimization is pointed out [25]. With the addition of renewable energy and other highly random objects, Zheng et al. adopt a stochastic modeling method for the unit commitment problem, propose the corresponding optimization tools, and also optimize the solution [26]. In addition to power system dispatching, there are some typical multi-objective complex optimization problems in other energy applications, such as a hybrid electric vehicle (hybrid electric vehicle, HEV) energy management. There are also a variety of operational objectives that need to be balanced and optimized. Yang et al. summarized the energy control problem of HEV and discussed the optimization of the energy management strategy in HEV [27]. In addition, Rodrigo et al. study the design of a heat exchanger with mixed integer complex optimization characteristics, which is also a typical multi-objective optimization problem in the field of energy, and obtained a wide range of attention and application attempts [28]. This paper analyzes the competition strategy of original equipment manufacturers and independent remanufacturers under the constraint of the carbon quota and trading policy and discusses the optimal competition strategy of original equipment manufacturers in the face of competition and environmental policy constraints of independent re-manufacturer by establishing a model.

In the application of multi-objective optimization, the traditional method is to convert it into a single-objective optimization problem by using weighting or linear programming methods, so it is difficult to select the weight, and the problem, with multiple conflicting objectives in practical engineering, cannot be effectively solved. With the continuous development of heuristic algorithms and their successful application to multi-objective optimization problems, a large number of multi-objective optimization algorithms are used in various industries.

In terms of research on portfolio strategies for low-carbon corporate development, studies by Fernando [29] and Cucchiella et al. [3] show that appropriate objective functions or a combination with other methods can make the application of the portfolio theory feasible in the area of power generation investment planning, which has been demonstrated in many studies related to the optimization of power structures. Kim et al. apply the Modern Portfolio Theory to construct efficient portfolios of different low-carbon technology fields. The empirical framework was applied to four countries (i.e., Germany, the United Kingdom, Italy, and France) for six low-carbon technology fields (i.e., renewable energy, smart grid, energy efficiency, sustainable transport, carbon capture and storage, and nuclear power) categorized [30]. Zhu et al. find that low-carbon economies has resulted in the innovative development of new energy sources that may persuade micro-investors to alter their investment behaviors. With this in mind, they proposed a dynamic quantitative trading system to assist investors in improving their new-energy sector profitability and better analyze the potential impacts of selected stocks [31]. The portfolio approach considers both the return and risk. When using the least cost approach, fossil energy generation technologies are given priority. The cost of fossil fuel power generation is often underestimated when external factors, such as environmental costs, are ignored. Therefore, the balance between low-carbon and -cost development needs to be considered jointly by businesses, governments and society, to maximize the cost and environmental benefits.

## 3. Model

As the basic economic organization of the society, enterprises are the backbone of realizing the double carbon. For the aim of double-carbon realization, enterprises should optimize the layout of industrial resource endowment characteristics and the introduction of advanced technology. Realizing intelligent service operation through the Internet of Things and digitalization is the way to achieve the development path of energy-saving and low carbon for enterprises. Previously, the theoretical research focused on developing a series of practical low-carbon strategies on the basis of technical support and the characteristics of enterprises. At present, there is little research on low-carbon transformation and a high-quality development path. Enterprises need to be powered through the three pillars, which are the energy system, economic system and ecosystem. According to the energy flow and material flow of the enterprise’s business activities, carbon neutrality is achieved from the energy system, economic system and ecosystem.

Based on the above problem description, the variables and parameters involved in this paper are described in Table 1.

### 3.1. Model Construction

Industry activities are full of optimization ideas. The portfolio strategy problem is an important branch of the optimization field, which can make companies and governments pay attention to the investment benefits of low-carbon transition. Investment is essentially choosing among uncertain paths. Companies should reduce the sensitivity of the existing energy economic system C, while the government improves the activity of their ecosystems E. This forms a multi-objective optimization problem as follows:

(1) $$\min C=C_{1}+C_{2}+C_{3}+C_{4},$$

(2) minE,

where $C_{1}$, $C_{2}$, $C_{3}$ and $C_{4}$ represent the carbon emission capacity of the existing energy system in power, energy use, fuel and raw materials, respectively. Reducing the sensitivity of the energy system is to improve the power decarbonization, energy electrification, fuel decarbonization and raw material decarbonization, respectively (see Table 2). 

Under this path, the general way for enterprises to achieve fuel decarbonization to improve $C_{1}$ includes using biomass waste as fuel to replace coal, using green hydrogen-derived synthetic fuel, such as biomass fuel, to replace petrol and kerosene, and replacing traditional natural gas or gas with green hydrogen and biomass natural gas as fuel, which is

(3) $$C_{1}=\sum_{j\in J}\gamma_{1j}x_{1j}+\sum_{j\in J}\delta_{1j}y_{1j}.$$

To achieve the decarbonization of raw materials $E_{2}$, enterprises usually use new energy vehicles rather than fuel truck in aspect of operating vehicles, commuting vehicles and logistics vehicles. Then we have

(4) $$C_{2}=\sum_{j\in J}\gamma_{2j}x_{2j}+\sum_{j\in J}\delta_{2j}y_{2j}.$$

To achieve fuel decarbonization $E_{3}$, companies have to reduce the amount of coal, petrol, kerosene, traditional natural gas, and gas, which is

(5) $$C_{3}=\sum_{j\in J}\gamma_{3j}x_{3j}+\sum_{j\in J}\delta_{3j}y_{3j}.$$

Finally, to achieve the decarbonization of raw materials $E_{4}$, that is to reduce the use of raw materials such as metals (steel and aluminum), petrochemical products (plastic, fiber, rubber), cement clinker and other raw materials, we have

(6) $$C_{4}=\sum_{j\in J}\gamma_{4j}x_{4j}+\sum_{j\in J}\delta_{4j}y_{4j}.$$

The total cost of the energy system for the company is

(7) $$C=\sum_{i\in I}\sum_{j\in J}\gamma_{ij}x_{ij}+\sum_{i\in I}\sum_{j\in J}\delta_{ij}y_{ij}.$$

The total emission of carbon dioxide among all production paths is

(8) $$E=\sum_{i\in I}^{}\sum_{j\in J}^{}\alpha_{ij}x_{ij}+\sum_{i\in I}^{}\sum_{j\in J}^{}\beta_{ij}y_{ij}.$$

There are three levels of restrictions for enterprises, which are the highest amount of investment, the highest requirements of carbon emission reduction, and the lowest amount of production capacities. The qualities of each path using the original production process can be considered as $x_{ij}$ and the qualities of each low-carbon path as $y_{ij}$ (see Table 2). 

The total investment can be considered as c and the government subsidy as s. Accordingly, the total investment is no more than the sum of the budget c and the subsidy s, which means $\sum_{i\in I}\sum_{j\in J}\gamma_{ij}x_{ij}+\sum_{i\in I}\sum_{j\in J}\delta_{ij}y_{ij}\leq c+s$. Let the carbon emission of the original production process $x_{ij}$ be $\alpha_{ij}$, carbon emission of low carbon path $y_{ij}$ be $\beta_{ij}$. Since the total carbon emission does not exceed e, we then have $\sum_{i\in I}^{}\sum_{j\in J}^{}\alpha_{ij}x_{ij}+\sum_{i\in I}^{}\sum_{j\in J}^{}\beta_{ij}y_{ij}\leq e$. The upgrade of low-carbon equipment needs investment. The total production capacities are more than the enterprise operation requirement p, which is $\sum_{i\in I}^{}\sum_{j\in J}^{}\varphi_{ij}x_{ij}+\sum_{i\in I}^{}\sum_{j\in J}^{}\eta_{ij}y_{ij}\geq p$. Therefore, we can establish the following multi-objective optimization model (I):

(9) $$(I)\;\;\;\;\;\;\min\left(C,E\right)=\left(\min C,\min E\right)$$

(10) $$s.t.\;\;\sum_{i\in I}\sum_{j\in J}\gamma_{ij}x_{ij}+\sum_{i\in I}\sum_{j\in J}\delta_{ij}y_{ij}\leq c+s,$$

(11) $$\sum_{i\in I}^{}\sum_{j\in J}^{}\alpha_{ij}x_{ij}+\sum_{i\in I}^{}\sum_{j\in J}^{}\beta_{ij}y_{ij}\leq e,$$

(12) $$\sum_{i\in I}^{}\sum_{j\in J}^{}\varphi_{ij}x_{ij}+\sum_{i\in I}^{}\sum_{j\in J}^{}\eta_{ij}y_{ij}\geq p,$$

(13) $$b_{ij}\leq x_{ij}\leq u_{ij},i\in I,j\in J,$$

(14) $${\bar{b}}_{ij}\leq y_{ij}\leq {\bar{u}}_{ij},i\in I,j\in J.$$

where (10) represents that the total cost is less than the sum of the cost budget for the company and the subsidy for the government, (11) represents that the total emission is less than the emission standards given by the government, (12) represents that the total production capacities are more than the enterprise operation requirement, and (13), as well as (14), represent the bounds of quantities on the original path and the low-carbon path, respectively. 

### 3.2. Model Formulation

The evaluation function is generally used in solving a multi-objective optimization problem. The basic idea of the evaluation function is to transform the multi-objective optimization problem into a single-objective optimization problem to solve. Moreover, the objective function of the single-objective optimization problem is constructed with the individual objective functions of the multi-objective problem.

In the model (I), we solve two single-objective optimization problems, respectively, as follows:

(15) $$\min C\;\;\;\;\;s.t.\;\left(10\right)-\left(14\right),$$

where $\left(x^{\prime },y^{\prime }\right)$ is the optimal solution. For the other single-objective optimization problem

(16) $$\min E\;\;\;\;\;s.t.\;\left(10\right)-\left(14\right),$$

$\left(x^{\prime \prime },y^{\prime \prime }\right)$ is the optimal solution. 

Then we have

$$C^{*}=\sum_{i\in I}\sum_{j\in J}\gamma_{ij}x^{*}{}_{ij}+\sum_{i\in I}\sum_{j\in J}\delta_{ij}y^{*}{}_{ij}.$$

Due to the contradictory thoughts of the multi-objective optimization problem, we then have $\left(C,E\right)\geq \left(C^{*},E^{*}\right)$, where ≥ in the sense of vectors, i.e., $C\geq C^{*}$ and $E\geq E^{*}$ in the usual numerical comparison. Obviously, $\left(C^{*},E^{*}\right)$ is an idea point that is almost impossible to reach. 

Then, the ideal point method is to find a point $\left(x^{*},y^{*}\right)$ in the feasible region of a multi-objective program, so that $\left(C\left(x^{*},y^{*}\right),E\left(x^{*},y^{*}\right)\right)$ is close to $\left(C^{*},E^{*}\right)$. That is, when the ideal point is known, the following single-objective optimization problem can be constructed under the measure ${\left\|\cdot \right\|}_{2}$.

(17) $$(\mathrm{II})\;\;\;\min F={\left\|\left(C,E\right)-\left(C^{*},E^{*}\right)\right\|}_{2}=\lambda \left|C\left(x,y\right)-C^{*}\right|+\mu \left|E\left(x,y\right)-E^{*}\right|\;s.t.\;\left(10\right)-\left(14\right).\;\;\;\;\;\;\;\;\;\;\;\;\;\;$$

The original multi-objective optimization model (I) is transformed into the single-objective optimization model (II). Besides, it is a completely different approach from the linear weighting method. The idea point method can equivalently deal with the nonlinear programming problem, while the linear weighting method can only equivalently deal with linear problems. 

## 4. Simulation Calculation

Studying different enterprises under the “dual-carbon” goal has different external constraints and internal capabilities. This paper divides enterprises into four sectors: primary resources, industrial manufacturing, public services and commercial consumption for simulation calculation. Some basic parameter settings are presented in Table 3. Some enterprise cases can be enumerated to derive some parameters. The quantities mentioned in Table 3. are comparative quantities based on the value of oil in the path O_12_. There are primary resource enterprises in China, such as the Tianqi Lithium Corporation (Chengdu, China), China National Offshore Oil Corporation (Beijing, China), Asia Pulp & Paper Co., Ltd. of Sinar Mas’ Group (Shanghai, China), and so on; industrial manufacturing enterprises, such as the China Baowu Steel Group Corporation (Shanghai, China), Anhui Conch Cement Co., Ltd. (Wuhu, China), LONGi Green Energy Technology Co., Ltd. (Xi’an, China), Saic Volkswagen Automotive Co., Ltd. (Shanghai, China), and so on; public service enterprises, such as the China Three Gorges Corporation (Wuhan, China), Alibaba Group Holding Limited (Hangzhou, China), Baidu (Beijing, China), Huawei Technologies Co., Ltd. (Shenzheng, China), Zhongxing Telecom Equipment (Shenzheng China), and so on. Representative enterprises in the commercial consumption area are Shui on Land (Shanghai, China), Ant Financial Services Group (Hangzhou, China), Beijing Science and Technology Co. (Beijing, China), Three Fast online (Beijing, China), and so on.

The primary resource sector, such as coal mine, oil, gas and energy production industry, faces risks of transformation. For example, valuation decline and sustainable goal-linked financing tools are needed to support its orderly transformation: using its mining resources, ecological restoration and new energy development provide clean energy, natural-based solutions and form negative emission business capabilities.

**Figure 1:**
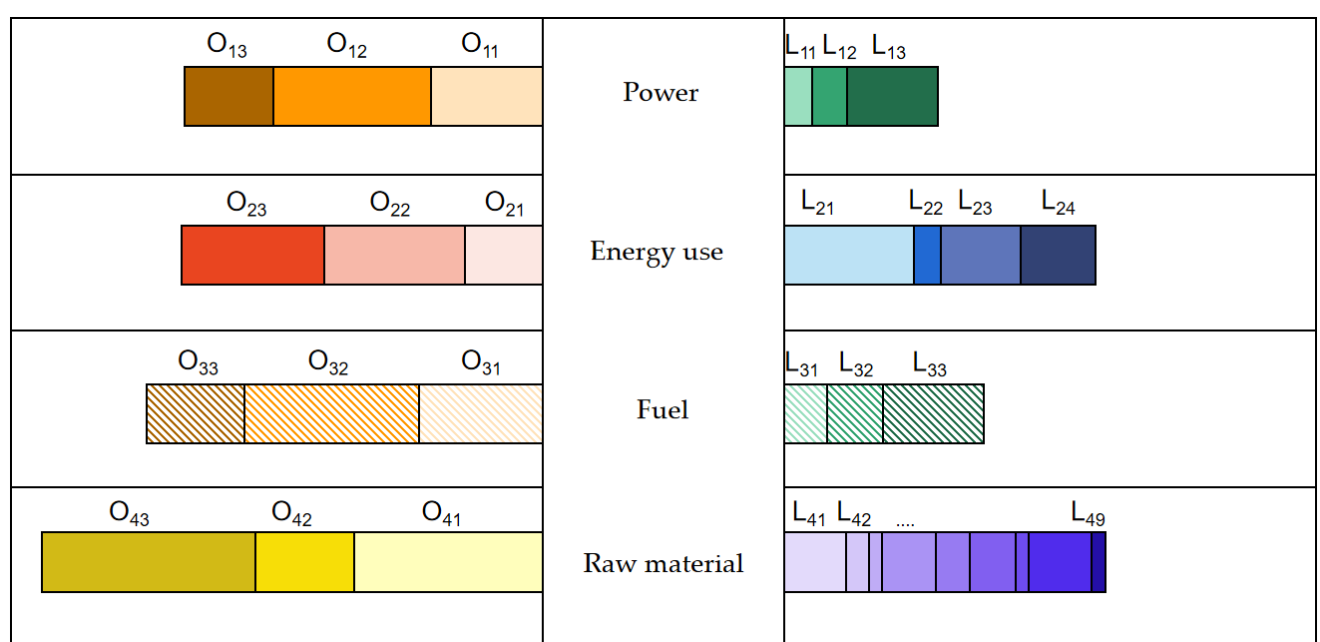
Low-carbon path transformation of primary resource enterprises.

Such enterprises need to accelerate their transformation, and the increase in government subsidies will greatly promote the transformation of enterprises from traditional development paths to low-carbon development paths, because of the high price of equipment renewal in the low-carbon development path, high technical thresholds, and low production capacity of some paths, as shown in Figure 1. The traditional development path is greatly affected by fuel prices, and the overall energy consumption level of the same traditional industrial production activity lags 2–3 times. Additionally, the fundamental problem lies in the industrial structure.

**Figure 2:**
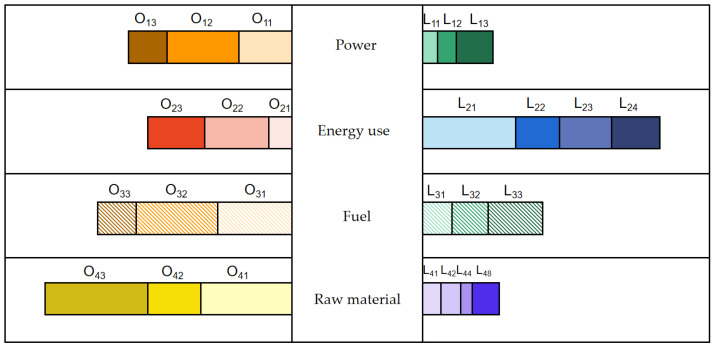
Low-carbon path transformation of the industrial manufacturing enterprises.

Opportunities and risks coexist in the industrial manufacturing sector. The material manufacturing has industry transformation risk. Some enterprise action reflects that the leading enterprises in the industry have means of electricity decarbonization, electrification, fuel decarbonization and negative carbonization, and realize the ability of orderly transformation, not only obtaining incremental market opportunities, but also exploring the stock market new opportunities through a low-carbon alternative. It is greatly affected by power, energy use and fuel, as shown in Figure 2.

**Figure 3:**
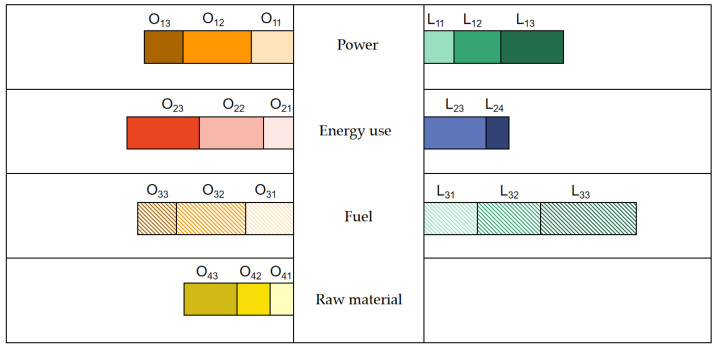
Low-carbon path transformation of the public service enterprises.

The public services sector is dominated by opportunities. Public services, especially in the power industry, have the dual dividend of electricity decarbonization and re-electrification, and the business opportunities will increase with the increasing proportion of electricity in terminal energy consumption. However, in order to carry a high proportion of renewable energy, it is necessary to continuously invest and participate in the construction of a new power system with new energy as the main body; however, risks and opportunities coexist. It is very affected by the power and fuel, as shown in Figure 3. 

By providing digital upgrading and digital and intelligent carbon reduction solutions in various industries, information technology has the opportunity to enter new markets in the low-carbon transformation. In the logistics and transportation industry, road freight, aviation and shipping industries are the industries with great difficulty in decarbonization technology. By adjusting assets and business structure, asset-light operation is stronger to avoid risks.

**Figure 4:**
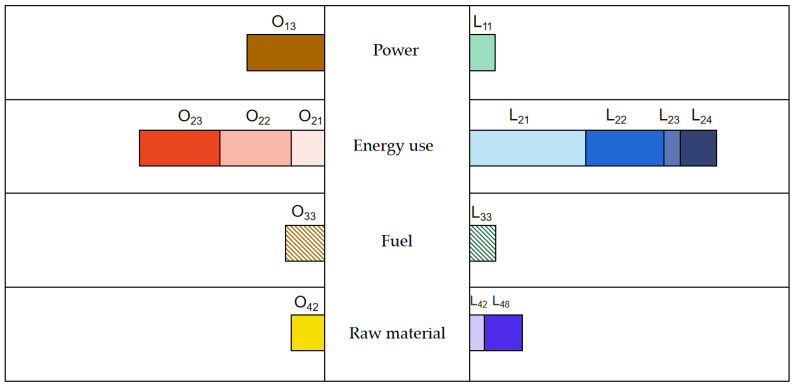
Low-carbon path transformation of the commercial consumption enterprises.

The commercial consumption sector presents a follower state, which is basically impregnable within an appropriate range, as shown in Figure 4. In the traditional business of the real-estate, textile and furniture, food and beverage industries, it is difficult to increase the market sales by borrowing the low-carbon transformation, or providing new selling points for users with low-carbon consumption preferences through differentiated positioning. Consumer Internet companies can help upstream products that already adopt low-carbon technologies and raw materials increase their market share through demand guidance.

## 5. Conclusions

The typical characteristic of the low-carbon economy is one that produces more with less. In other words, making the best use of things, using them comprehensively, recycling, and obtaining more, higher value added and more sustainable products and services with less energy resource consumption and environmental emissions. Energy cannot be recycled, but materials can be reused, and the essence is to improve resource efficiency. The goal of carbon peaking and carbon neutrality imposes new requirements on energy and fuel use.

There are four types of enterprises, and each type has its own path to low-carbon transition. As for the primary resource sector, such as coal mine, oil, gas and energy production industry, it faces risks of transformation. Due to a valuation decline, sustainable goal-linked financing tools are needed to support its orderly transformation. The increase in government subsidies will greatly promote the transformation, because of the high price of equipment renewal in the low-carbon development path, high technical thresholds, and low production capacity of some paths. The traditional development path is greatly affected by fuel prices, and the fundamental problem lies in the industrial structure.

Opportunities and risks coexist in the industrial manufacturing sector. The material manufacturing has industry transformation risk. Some enterprise action reflects that the leading enterprises in the industry have the means of electricity decarbonization, electrification, fuel decarbonization and negative carbonization, realize the ability of orderly transformation, not only obtaining incremental market opportunities, but also exploring stock market new opportunities through a low-carbon alternative.

Adjusting assets and business structure, asset-light operation and information technology in the public services sector are both stronger than the logistics and transportation industry, to avoid risks. This especially regard the dual dividend of electricity decarbonization and re-electrification in the power industry, as its business opportunities will increase with the increasing proportion of electricity in terminal energy consumption. Last, but not least, the commercial consumption sector presents a follower state, which is basically impregnable within an appropriate range. In the traditional business of the real-estate, textile and furniture, food and beverage industries, it is difficult to increase the market sales by borrowing the low-carbon transformation.

There are some suggestions of reducing the carbon footprint to achieve low-carbon transformation. Enterprises should promote supply chain low-carbon plans by requiring suppliers to join common carbon reduction targets and providing carbon reduction support programs. Then, it is significant to implement internal carbon pricing by pricing carbon emissions for each business unit and charging a fee. Applying green sustainable finance tools to drive transformation is also an efficient way. Furthermore, establish innovation alliances to obtain the technical capabilities required for carbon reduction of inherent businesses is important. Reducing the carbon footprint can be achieved through other methods, such as the optimization of business structure and optimizing the product or service design. There are some measures on enlarging the carbon handprint. Enterprises should promote the development of low-carbon/carbon-negative technologies and alternatives. It is especially necessary to provide scenario-based carbon reduction solutions and green finance support, which can guide demand and behavior change.

Carbon neutrality has provided clear indicators and goals for the high-quality development of China’s economy in the future years. The low-carbon transformation of enterprises needs to rely on technological innovation. For technological innovation enterprises, the “double-carbon” goal has spawned a large number of carbon reduction needs in traditional industries and provided a rare application scenario. For traditional enterprises, the low-carbon transformation integrates the boundaries of different industries, breaking the existing market pattern and creating new drivers.

## Figures and Tables

**Table 1:** Description of symbol.

Symbols	Explanation
i∈I	Energy system index set
j∈J	Path index set (including original paths and low-carbon paths)
$\alpha_{ij}$	Unit emission of each original path
$\beta_{ij}$	Unit emission of each low-carbon path
$\gamma_{ij}$	Unit cost of each original path
$\delta_{ij}$	Unit cost of each low-carbon path
$\varphi_{ij}$	Unit production capacity of each original path
$\eta_{ij}$	Unit production capacity of each low-carbon path
$x_{ij}$	Quantity of each original path
$y_{ij}$	Quantity of each low-carbon path

**Table 2:** Development path of electricity, energy use, fuel and raw materials.

Energy System	Path Property	Specific Path (Path Code)	Quantity
Power	The original path	Coal as a fuel in the production of electric energy (O_11_)	$x_{11}$
		Oil as a fuel in the production of electric energy (O_12_)	$x_{12}$
		Natural gas as a fuel in the production of electric energy (O_13_)	$x_{13}$
	Low-carbon path	Use biomass waste (L_11_)	$y_{11}$
		Use biomass fuel oil and other green hydrogen-derived synthetic fuel (L_12_)	$y_{12}$
		Use green hydrogen and biomass natural gas (L_13_)	$y_{13}$
Energy use	The original path	Operating, commuter, and logistics vehicles (O_21_)	$x_{21}$
		Construction machinery process (O_22_)	$x_{22}$
		Heat addition (O_23_)	$x_{23}$
	Low-carbon path	Operating vehicles, commuter vehicles and logistics transport vehicles adopt new energy vehicles (L_21_)	$y_{21}$
		Construction machinery and vehicles are powered by hydrogen fuel or pure electricity (L_22_)	$y_{22}$
		The airport provides ground power supply, and the terminal provides shore power (L_23_)	$y_{23}$
		Use electric boiler and heat pump technology for heating; industrial process electrification transformation (L_24_)	$y_{24}$
Fuel	The original path	Coal (O_31_)	$x_{31}$
		Gasoline, kerosene (O_32_)	$x_{32}$
		Traditional natural gas, coal gas (O_33_)	$x_{33}$
	Low-carbon path	Use biomass waste as a fuel (L_31_)	$y_{31}$
		Use biomass fuel oil, green ammonia, green methanol and other green hydrogen-derived synthetic fuels (L_32_)	$y_{32}$
		Use green hydrogen, biomass natural gas as fuel (L_33_)	$y_{33}$
Raw material	The original path	Metals (steel and aluminum) (O_41_)	$x_{41}$
		Petrochemical products (plastics, fiber, rubber) (O_42_)	$x_{42}$
		Cement clinker (O_43_)	$x_{43}$
	Low-carbon path	Use recycled batteries to produce electrode materials, recycle metal raw materials, and recycle non-metallic materials (L_41_)	$y_{41}$
		Use plant-based packaging, plant-based dye, and plant-based fibers (L_42_)	$y_{42}$
		Catch carbon dioxide as a raw material for synthetic chemical raw materials (L_43_)	$y_{43}$
		PET and other plastic recycled raw materials (L_44_)	$y_{44}$
		Slag instead of clinker or raw material (L_45_)	$y_{45}$
		Green electric metal raw materials (L_46_)	$y_{46}$
		Green hydrogen as the raw material (L_47_)	$y_{47}$
		Recycled raw materials for packaging material (L_48_)	$y_{48}$
		Plant protein raw materials (L_49_)	$y_{49}$

**Table 3:** Parameter settings (comparative quantity).

Energy System	Path Code	$\alpha_{ij}$	$\beta_{ij}$	$\gamma_{ij}$	$\delta_{ij}$	$\varphi_{ij}$	$\eta_{ij}$
Power	O_11_	15		6		8	
	O_12_	10		10		10	
	O_13_	7		20		14	
	L_11_		5		2		4
	L_12_		4		2		8
	L_13_		2		6		14
Energy use	O_21_	8		12		8	
	O_22_	9		12		15	
	O_23_	15		6		15	
	L_21_		1		12		8
	L_22_		1		16		15
	L_23_		1		18		15
	L_24_		5		18		8
Fuel	O_31_	15		6		8	
	O_32_	8		3		10	
	O_33_	7		20		14	
	L_31_		5		2		4
	L_32_		4		2		8
	L_33_		2		6		10
Raw material	O_41_	5		10		6	
	O_42_	5		30		8	
	O_43_	4		2		8	
	L_41_		5		5		8
	L_42_		4		6		6
	L_43_		1		22		4
	L_44_		5		19		5
	L_45_		4		62		5
	L_46_		3		84		1
	L_47_		1		6		6
	L_48_		4		3		3
	L_49_		3		30		5

## Data Availability

Not applicable.
